# Balancing protection of participants and other stakeholders with openness: African lessons from the MADIVA data sharing and access policy

**DOI:** 10.1080/16549716.2025.2590307

**Published:** 2025-12-16

**Authors:** Daphine Tinashe Nyachowe, Victoria Bronstein, Helen Robertson, Scott Hazelhurst, Jillian Gardner, Michele Ramsay, Patrick Opiyo Owili, Michael Klipin, Kerry Glover, Gershim Asiki, Henry Owoko Odero, Phyllis Mungai, Diana Awuor, Samuel Iddi, Isaac Kisiangani, Daniel Maina Nderitu, Nkosinathi Masilela, Daniel Ohene-Kwofie, Faith Kimongo, as members of the MADIVA Research Hub and the DS- I Africa Consortium

**Affiliations:** a Sydney Brenner Institute for Molecular Bioscience, Faculty of Health Sciences, University of the Witwatersrand, Johannesburg, South Africa; b School of Law, University of the Witwatersrand, Johannesburg, South Africa; c School of Computer Science and Applied Mathematics, University of the Witwatersrand, Johannesburg, South Africa; d School of Electrical and Information Engineering, Faculty of Engineering and the Built Environment, University of the Witwatersrand, Johannesburg, South Africa; e Steve Biko Centre for Bioethics, School of Clinical Medicine, Faculty of Health Sciences, University of the Witwatersrand, Johannesburg, South Africa; f Faculty of Health Sciences, University of the Witwatersrand, Johannesburg, South Africa; g African Population and Health Research Center (APHRC), Nairobi, Kenya; h MRC/Wits Rural Public Health and Health Transitions Research Unit, University of the Witwatersrand, Johannesburg, South Africa

**Keywords:** data sharing, data sharing policies, LMICs, stakeholders, multimorbidity

## Abstract

National and international data sharing in health research is critical for advancing scientific discovery and fostering collaborative partnerships. With growing technological advances and an increasing desire for open science, data sharing enables researchers to access diverse datasets to generate novel insights. Adopting robust policies is key to responsible data sharing, which fosters interdisciplinary collaboration, ensures ethical and legal compliance, implements transparency, and strengthens stakeholder trust. Collaboration is increasingly important for health research, and therefore, effective data sharing policies are indispensable in balancing competing interests that arise in the complex research environment. This paper shares insights from Multimorbidity in Africa: Digital Innovation, Visualisation, and Application (MADIVA), a project that explores multimorbidity in African populations. In addition to facing typical data sharing challenges, the hub confronts specific challenges prevalent in low- and middle-income settings. MADIVA’s data access and sharing policy balances the competing demands of open science while safeguarding the interests of researchers and participants. This case study offers a framework for data sharing in collaborative health research across resource-constrained settings that can be adapted for use by others.

## PAPER CONTEXT

**Main findings:** This paper highlights the importance and need to balance various stakeholder interests in data sharing policies to ensure equitable data sharing.

**Added knowledge:** This paper presents insights from the MADIVA research hub on developing a data access and sharing policy in a low- and middle-income setting that balances participant and community protection, open science, and stakeholder interests.

**Global health impact for policy and action:** The paper aims to encourage health research projects particularly those based in low- and middle-income countries to develop data sharing policies in order promote equitable data access and strengthen collaborations.

## Background

This millennium has seen an increase in data sharing across the globe. Increased technological advances allow data to be accessed and processed quickly across jurisdictions. These technological innovations substantially benefit health research and systems by identifying and bridging gaps in research, fostering collaboration, and supporting scientific discovery [1]. At the same time, the emergence of new technologies has created and/or exacerbated difficulties with sharing health data. These challenges include privacy and confidentiality concerns, which are centred on ensuring the proper protection of participants’ data, ethical and legal dilemmas and/or data security barriers, data storage and flow intricacies across jurisdictions and resource limitations such as funding and expertise in data management. In the face of these benefits and challenges, researchers must ensure that data is responsibly shared. This implies that the need to share data for research and innovation must be carefully balanced by ethical and legal considerations. The implementation of a data-sharing policy is a strategy that can assist in achieving this balance. Effective data sharing policies define who can access data, under what conditions, how it can be shared, and for what purposes [2]. Organisations transparently lay down data sharing practices to ensure that data will be shared responsibly.

The process of collecting data is done in an ecosystem involving tools, technologies, stakeholders, and workflows. Data collection requires resources and is typically conducted by researchers after the study has been designed, and ethical approval has been received. *Primary research data* is traditionally collected from participants or data subjects based on a specific hypothesis. Clinical data is also primary data that was not originally collected for research purposes. It has become increasingly common for researchers to reuse primary data that was previously collected for a different purpose. Such data is known as s*econdary data*. Researchers are required to protect both primary and secondary data and ensure that participants’ data is used in a way that conforms to legal and ethical obligations. These obligations affect the interests of all those involved. After several engagements, the MADIVA team identified that the most salient interests are: (1) the protection of participants and communities, (2) the advancement of open science, and (3) the protection of other stakeholders including but not limited to researchers and funders.

**Figure 1.**
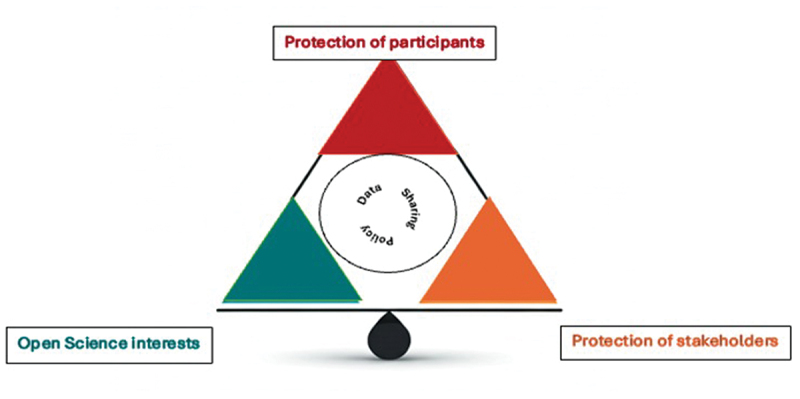
Balancing competing interests in a data sharing policy.

Balancing the three categories of interests is the key to a responsible data access and sharing policy. In the first corner of the triangle in Figure 1, participants and communities need to be safeguarded to ensure that they are treated with respect and dignity while being protected from harm that may arise from the use of their data [3]. Participants and communities face the risk of potentially being identified by data intruders with access to other data bases [3]. Once a participant has been identified, the information can be used by insurance companies, employers or others to discriminate against them [3]. There can be a risk of stigmatisation. These risks are particularly salient in indigenous communities and CARE (Collective Benefit, Authority to Control, Responsibility, Ethics) principles have been developed to protect their interests. It is particularly important that participants, families, and communities that contribute data share in the rewards that come from scientific development [4,5]. Data sharing policies enforce transparency by clearly defining the study’s approach to data sharing and protecting participants and communities. A data sharing policy should articulate how sensitive information will be protected during the data sharing process. To uphold this transparency, data sharing policies should be publicly available. Another dimension is data security given that much of the data is very sensitive, and security breaches or ransomware attacks are very serious threats. Precautionary measures should always be put in place to secure data. However, this is beyond the scope of this paper.

The second corner of the triangle represents the promotion of scientific integrity, reproducibility, and progress through the advancement of open science. This reflects the need to make scientific research and its dissemination transparent and accessible [6]. Adherence to FAIR principles, which require data to be Findable, Accessible, Interoperable, and Reusable supports this goal [7]. Scientific knowledge should be ‘as open as possible’ [8] to provide palpable benefits and accelerate progress through collaboration. Timely data sharing benefits the research community. It is also important to recognise that the costs of collecting data are high in resource poor communities where an ethical imperative exists to maximise the value of participants’ data. There is generally a need for high quality data in LMICs to improve health outcomes [9]. It is also fundamental that those individuals who collect or aggregate data store it securely and share it responsibly to ensure long-term sustainable access and sharing [10].

The third corner of the triangle represents protection of stakeholders apart from participants and communities (who are also stakeholders). In this paper, we use the term ‘stakeholders’ to mean individuals and organisations that directly or indirectly invested resources into the data collection and research process. Typically, these stakeholders include co-investigators, collaborators, government, and funding agencies. It is meaningful that institutions that collect data in Low- and Middle-Income Countries (LMICs) remain sustainable so that the communities that they serve continue to be represented in scientific research.

It is important to consider the interests of researchers and institutions that collect and analyse data when formulating a robust data sharing policy. To some extent, researchers may have to balance the greater good above their individual goals in such scenarios. Data sharing policies are guiding tools for achieving this balance. These policies often promote and encourage data sharing, reproducibility of scientific knowledge, and data reuse, while disclosing how this will be achieved without compromising other interests. For example, a data sharing policy might declare that data will be made publicly available or available to a particular category of individuals (for example, *bona fide* researchers) while setting out the restrictions and processes for sharing the data.

A data sharing policy should give meaningful protection to all the stakeholders. Achieving this goal is particularly challenging because, unlike the objectives of protecting participants and advancing open science, protecting stakeholders is a multi-dimensional endeavour. This is due to the presence of multiple stakeholders, each with their own unique set of diverse interests. While researchers aim to conduct novel research and advance their career goals, funders also need assurance that their funds are utilised in line with their objectives or priorities. Researchers and funders need to operate within the framework of national laws and ethics requirements. This is to ensure that everyone’s interests are considered. Data sharing policies create conditions that set out how these interests are balanced [2].

Data sharing policies are crucial in LMICs, where they ensure that data sharing is achieved in a manner that does not disadvantage or harm participants, communities, researchers, or research institutions [11]. LMICs experience different and significant challenges when handling and sharing data (more so when handling genomic data) from those arising in high-income settings [1,12,13]. Institutions in LMICs are often weaker and more fragile than those in developed countries and LMIC researchers have justifiable fears of being scooped. They also suffer from a ‘lack of coherent national policies’, and ‘poor research infrastructure’ [14]. Data sharing policies need to be enabling mechanisms that foster sustainable research in LMIC institutions. In other words, policies must not undermine the capacity of LMIC institutions to contribute high-impact research from start to finish especially when they choose to engage with international collaborators. Data sharing policies must enable research groups in LMICs to remain sustainable and continue to be attractive recipients of research funding into the future. There is a need to develop ethical and sustainable data sharing systems that do not disadvantage the researchers and institutions that operate in these settings. It is necessary that those who collect or aggregate data in these complicated research environments have a fair opportunity to analyse their own data before having to share it with other researchers [15]. Although timely data sharing may be vital in LMICs, especially when dealing with urgent situations such as pandemics or epidemics, it is essential to do so equitably without undermining their data rights [16].

A data sharing policy that balances the interests of participants, open-science and stakeholders successfully achieves responsible data sharing. MADIVA recognised the need to balance these interests and developed its data access and sharing policy (MADIVA policy). To our knowledge, the MADIVA policy (which can be accessed here) was the first one to be developed in the Data Science for Health Discovery and Innovation in Africa (DS-I Africa) consortium. Hence, the hub was required to be thorough and resourceful, using existing policies within the field and from collaborators.

## MADIVA case study

MADIVA is a research hub of the DS-I Africa initiative. DS-I Africa aims to leverage data science technologies to transform biomedical and behavioural research and ultimately develop solutions that lead to improved health for individuals and populations in Africa and the world [17]. MADIVA is developing data science techniques and solutions for tackling multimorbidity in Africa [18]. *Multimorbidity* is defined as the presence of two or more chronic diseases in an individual such as diabetes, hypertension, obesity, and human immunodeficiency virus infection [19,20]. Multimorbidity is a serious global health problem that significantly adds to the health burden, particularly in Africa, where resources are often limited. African insights will be able to enhance the academic literature so that the treatment of multimorbidity can be optimised in any health context where resources are limited. To achieve its objectives, the hub utilises secondary data from two primary research sites: rural Bushbuckridge (Agincourt), South Africa, and two urban informal settlements of Nairobi, Kenya, collected by the African Population and Health Research Center [21]. Both sites have rich datasets from longitudinal studies dating back as far as the year 2003 from 20,000 households in Agincourt and 33,463 households in Nairobi, gathered by health and demographic surveillance systems and nested research studies over decades. These records include demographic data, clinical health records, and genomic data which are valuable for this study. Genomic data is particularly valuable to this study because it contributes to understanding the influence of genetic factors on multimorbidity in these populations.

MADIVA activities include developing and applying data science techniques to (a) link different datasets; (b) build dashboards for health workers, managers, and departments to assist them in managing multimorbidity in their respective communities; (c) promote public precision health by developing risk prediction models, including the use of automatic stratification to identify high-risk sub-populations and polygenic scores to understand the heritable components of diseases. The outcomes of this project will assist in improving the management of multimorbidity, especially in South Africa and Kenya, with the hope of extending it to other LMICs and the rest of the world.

MADIVA is a collaborative effort that requires data to be shared and accessed between different parties, often across jurisdictions. The MADIVA project presents numerous complexities that make data sharing both essential and challenging. These intricacies highlighted the urgency of establishing a clear and effective policy to facilitate secure and efficient data sharing.

### The making of the policy

Establishing a data access and sharing policy was a priority at the commencement of the project. However, the process took over 2 years, reflecting the complexity and need for broad stakeholder engagement. MADIVA’s Training, Capacity Development and Pilot Core (TCDPC) and Data Management and Access Committee (DMAC) met with various stakeholders, including community leaders acting on behalf of participants, data contributors, policymakers, and health officials to discuss aspirations and concerns regarding the outcome of the project. The information from these discussions was used to determine how each stakeholder’s interests would be protected while ensuring that data would be adequately shared for the benefit of the project and participant communities.

**Figure 2.**
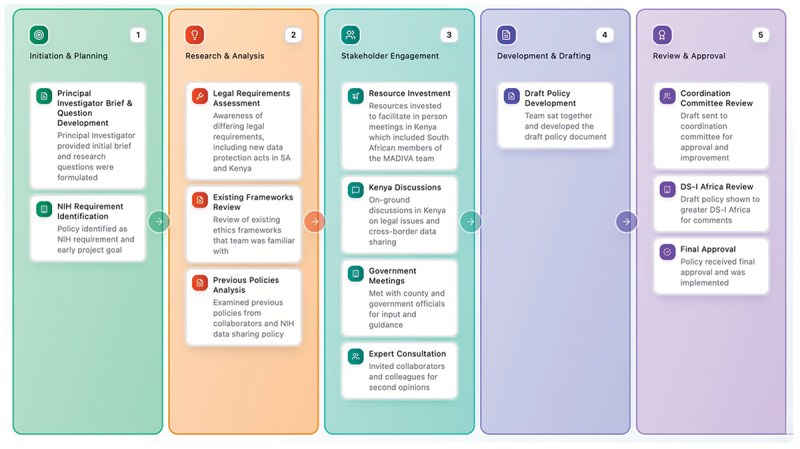
MADIVA policy development process.

At the time of initiating this policy, South Africa and Kenya had recently implemented data protection laws (South Africa’s Protection of Personal Information Act 4 of 2013 [22] and Kenya’s Data Protection Act of 2019 [23], respectively) that required interpretation to understand their application to health research. In July 2023, a broad range of members of the MADIVA team from South Africa and Kenya met in Nairobi over a week to determine the implications of the new regulations for our research. The starting point was to identify the factual details relevant to the MADIVA data such as the types and kinds of data, the overall data sharing pipeline, the extent to which the data could be deidentified, the sensitivity of the data, and data storage. An analysis of the legal and ethical implications of data sharing requires a thorough understanding of the data. The team included lawyers, a philosopher/ethicist, data governance experts, data scientists, biostatistician and public health experts, etc. A key takeaway from this experience is that policymaking of this nature is interdisciplinary that requires meaningful engagement and involvement of different expertise. After that, the adoption of the policy took well over a year, partly due to disagreements within our team over how to resolve the tension between open science and the protection of stakeholders. This was the start of an iterative process which continuously improved the policy. There were several meetings, discussions, and compromises. What made the process successful in the end was ensuring that everyone understood the issues and the viewpoints of others, and a deliberate decision not to rush the process to allow the issues to be debated until a consensus could be reached. In our case, initial disagreements did not form around institution or country lines but rather tended to be based on the role of researcher (e.g. data scientist, data collector). The primary reasons for this are that we agreed early on that each organisation would be custodian of its own data in order to lay a foundation for building trust and to develop an integrated team. A clear understanding of the legal constraints that country-specific laws created also helped this process. The policy development process is summarised in Figure 2.

## Overview of the MADIVA data access and sharing policy

The MADIVA data access and sharing policy aims to maximise the availability of the data collected by the hub by adopting FAIR principles. Data needs to be made available to *bona fide* researchers in a timely and reasonable manner, with due consideration of participants’ rights. (Researchers will still be required to obtain the necessary ethics approval to process the data in the particular country or countries). At the same time, the policy recognises the importance of equitable data sharing by allowing members of the MADIVA hub exclusive access to their data for a limited and reasonable time.

Balancing the principles of open science with periods of exclusivity for hub members addresses tensions that are particularly acute in LMICs. For a project such as MADIVA, which has rich and complex data, it is crucial to protect and strengthen local research capacity, reduce power imbalances in international collaboration, ensure ethical and fair data governance, and prevent exploitation by well-resourced international parties. MADIVA members must have a fair opportunity to use and publish their own data without being scooped by others. This is why the policy incorporates an embargo period to protect the interests of researchers. This also provides scientific advantages because hub researchers that understand the social context in which the demographic and health data was collected will generate fewer errors when analysing and interpreting the data and produce better results.

The MADIVA policy envisages data access and sharing by different categories of parties:

- Members of the MADIVA team during the MADIVA research phase.
- External collaborators of MADIVA while the MADIVA hub remains active.
- Non-collaborative sharing including after the MADIVA hub is no longer active.

### Members of the MADIVA team during the MADIVA research phase

MADIVA research hub processes de-identified secondary data, in the sense that primary personal identifiers have been removed from the data and location data has been re-coded to allow researchers to establish the geographical proximity of data subjects to one another without revealing actual locations. Further, the data is deidentified in MADIVA’s hands so the link to participants is lost. Despite these efforts, the personal data remains sensitive as reidentification may be possible. The data sharing policy establishes several conditions of access including requiring members of the MADIVA team to undertake to follow secure computing practices such as using the data on the Wits Core Research Cluster. This ensures that a researcher’s processing and data transfer is audited and leaves a trail that could be inspected. Hub members also need to sign a confidentiality pledge where they undertake not to attempt to ascertain the identity of any data participant or allow anyone else to do so. According to the policy, the same pledge will be necessary for any collaborators or external users of the data in future. The inclusion of these measures is essential for longitudinal studies that may require medical referral.

The data sharing policy establishes mechanisms, procedures, and conditions of access and use during the project term. This is essential because it creates boundaries and processes that assist in tracing events in which the data is misused. MADIVA researchers may not share data with anyone outside the hub unless the outsiders have obtained express written approval from the Coordination Committee (CC). While most of the provisions that apply to the MADIVA team are directed at protecting participants, some provisions protect other stakeholders, including researchers.

**Figure 3.**
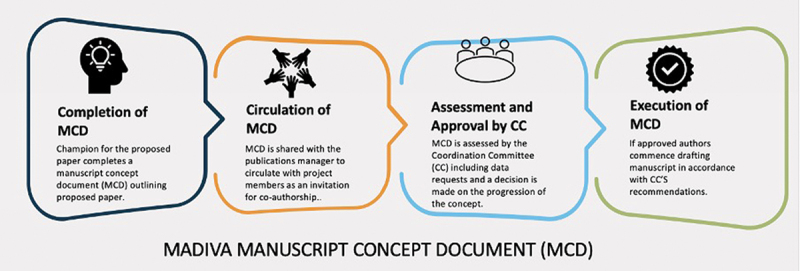
MADIVA manuscript concept document procedure.

The policy balances competing interests within MADIVA by including a publication policy which aims to proactively deal with overlaps in the work that MADIVA researchers are doing. Those wishing to publish using MADIVA data must first submit a Manuscript Concept Document (MCD) outlining the proposed research. The MCD is circulated among the members of MADIVA who are given an opportunity to contribute to the paper. Ultimately, the CC approves the MCD which must comply with MADIVA’s publication policy. For example, the CC will avoid overlap and unhealthy competition between members of the Hub. MCDs need to be updated if there is a change in scope. This process is represented below in Figure 3. Finally, researchers are required to acknowledge the funders in their publications. These acknowledgements build accountability as they allow funders to track their contribution to both data collection and science.

### External collaborators of MADIVA while the hub remains active

MADIVA contributes to an elaborate metadata catalogue that is held by eLwazi and was developed by data stewards who were appointed by each DS-I Africa Hub. (‘eLwazi is an African led open data science platform that provides an interactive environment to apply data science techniques to diverse datasets for novel health discoveries’) [24]. The data stewards developed the metadata catalogue and continue to keep it updated. This enables external parties to know what data is available in MADIVA and how it can be accessed.

External parties who wish to collaborate with MADIVA submit a data request form to the CC. The CC considers requests on a case-by-case basis, considering factors such as privacy protection of participants, benefit-sharing agreements including potential co-authorships, scientific quality, ethical and social risks, and benefits for long-term collaboration. There are special principles and procedures that are required for further sharing of genomic data that is in the custody of MADIVA. This is because the genomic data is subject to broad consents that were given ethics approval on condition that the original research ethics committee be re-approached before the genomic data be used for other projects. This is in addition to usual research ethics approval required by future researchers. The MADIVA policy provides protection for stakeholders as every institution that contributes data to the MADIVA hub is represented on the CC when the decision to share data is made,

Once the CC gives consent and the external party has the necessary ethics approval, a data transfer agreement is entered into with the recipient of the data. MADIVA is open to making its data available subject to key terms and conditions. In such projects, it is easy to share data remotely; therefore, clauses are put in place to ensure continued ethical and legal compliance by the recipients of the data. MADIVA recognises that collaborations with external parties can be beneficial, and this procedure is available for that purpose.

The main objective of this part of the MADIVA policy is to protect participants while advancing scientific endeavours; however, the decision-making structure ensures that stakeholders’ interests are also considered in the process. Therefore, these measures protect participants, their data, and stakeholders who contributed data. Hence, we see the three competing interests that are depicted in Figure 1 carefully balanced within the policy.

### Non-collaborative sharing including after the MADIVA hub is no longer active

MADIVA is committed to making data as accessible as possible, including to non-collaborators even after completion of the project. This is also in line with the far-sighted policies of the NIH in the DS-I Africa Consortium which focuses on building a repository or repositories of quality African data to fill gaps in the global health research enterprise. The commitment to open science does not mean that the obligation to keep the data safe ceases after the project funding cycle comes to an end. The CC will decide when data will be released to a data repository or data repositories. The data will normally be released within 24 months, with a further 12 months publishing embargo.

The details of data storage after the project has terminated are not yet fully fleshed out in the policy. Consequently, development of the policy will be necessary in future. The current situation is that the most appropriate repositories for long-term storage of the data will be identified depending on the nature of the data. These repositories are likely to be located at institutions that contributed the data. The European Genome-Phenome Archive [25] and the African Population and Health Research Center Microdata-Portal have been approved as repositories for continued storage of MADIVA data and other repositories may be approved.

The composition of the Institutional Data Access Committees that will control access to the data have not yet been determined and will depend on where the data is stored. The policy does, however, contain a well-developed set of criteria for Data Access Committees to consider before sharing the data. These emphasise guarantees for protection of participants and consider the scientific merit of the proposal, the capacity of the team, and whether African scientists will be included as collaborators by the requesters.

## Lessons learned

MADIVA is funded by the NIH and the data sharing policy was a requirement of the grant. This process of developing a data sharing and access policy illustrates the advantage of having a funder that pays close attention to data governance so that the ethical and legal responsibilities of researchers are emphasised and the data is used to its full potential. It is important that funders ensure that the projects they fund pay proper attention to these matters. It is also important that projects like MADIVA have diverse, multidisciplinary teams with sufficient legal and ethical expertise. The communication between the technical, legal, and ethical members of the team needs to be excellent and there needs to be dedicated time where these matters are prioritised. There are substantial benefits of a good transparent data sharing policy as it improves the relationship of members of the team and relationships with outside researchers.

## Conclusion

MADIVA’s emphasis on multimorbidity means that its data focuses on health problems which will continue to become more prevalent. MADIVA data could potentially assist with the management of these conditions. The genomic data in the project offers a valuable opportunity for advances in healthcare but also requires rigorous engagement with far-reaching ethical and legal challenges associated with sharing this type of data in the African context. These include population-specific genetic variations, potential for stigmatisation, and the importance of community engagement in genomic research. The MADIVA policy strives to promote the aspiration of open science without compromising the safety of participants and communities.

The MADIVA policy establishes a workable balance between protection of participants and communities, support for scientific discovery through open science and protection of other stakeholders. The policy establishes clear guidelines for data management during and after completion of the project. This ensures that data remains available for research and innovation after the project ends, while safeguarding participants’ rights and privacy. The mechanisms implemented in this policy also promote equitable and responsible data sharing practices that provide projects like MADIVA and its researchers with an opportunity to flourish.

The MADIVA policy has emerged from a participative process which involved deep engagement with the ethical and legal aspects of processing secondary health data. Critical lessons for the successful adoption were: (a) ensuring that there was firm understanding of the ethical and legal issues involved; (b) building a culture of trust within the collaboration; (c) establishing a process which allowed meaningful debate and discussion. By not rushing the process, we allowed everyone the opportunity to understand others’ viewpoints and to shift their position over time. Although not an issue in our case, other collaborations may find that disagreements are institutionally based, and we think the same processes would be useful to others.

The policy is also useful as it provides a model for the governance of complex research projects which aggregate data from multiple sources. Critical analysis of previous policies is invaluable for other projects that aspire to develop their own policies for governance of secondary data in health research. The MADIVA policy establishes formal structures for managing large, interest-sensitive collaborations, empowers LMIC researchers to lead with their own data, and promotes collaboration through transparency and a metadata catalogue.

Moving forward, continuous evaluation and adaptation of the data access and sharing policy will be essential to address emerging challenges in data governance and regulations, technological advancement, and evolving ethical standards. By doing so, the policy will maintain a balance of the competing interests discussed in this article and reflected in Figure 1. In sharing this policy, we hope to assist in developing debates which will enhance the development of data sharing policies.

## Data Availability

Data sharing is not applicable to this article as no data were created or analysed in this study.

## AI tools

Figures 1 and 2 were developed using online AI tools, Google AI image generator and Figma, respectively.

## References

[1] Bull S, Roberts N, Parker M. Views of ethical best practices in sharing individual-level data from medical and public health research: a systematic scoping review. J Empir Res Hum Res Ethics. 2015;10:225–10. doi: 10.1177/1556264615594767 26297745 PMC4548478

[2] Tan AC, Webster AC, Libesman S, et al. Data sharing policies across health research globally: cross‐sectional meta‐research study. Res Synth Methods. 2024;15:1060–1071. doi: 10.1002/jrsm.1757 39275943

[3] Heeney C, Hawkins N, de Vries J, et al. Assessing the privacy risks of data sharing in genomics. Public Health Genomics. 2010;14:18–19. 10.1159/000294150 PMC2872768 20339285

[4] Amorim M, Silva S, Machado H, et al. Benefits and risks of sharing genomic data for research: comparing the views of rare disease patients, informal carers and healthcare professionals. Int J Environ Res Public Health. 2022;19:8788. doi: 10.3390/ijerph19148788 35886636 PMC9319916

[5] Carroll SR, Garba I, Figueroa-Rodríguez OL, et al. The care principles for Indigenous data governance. Data Sci J. 2020;19:43. doi: 10.5334/dsj-2020-043

[6] UNESCO. Recommendation on Open Science [Internet]. Geneva: World Health Organization. [cited 2025 Mar 28]. Available from: 10.54677/MNMH8546

[7] Wilkinson M, Dumontier M, Aalbersberg I, et al. The fair guiding principles for scientific data management and stewardship. Sci Data. 2016;3:160018. doi: 10.1038/sdata.2016.18 26978244 PMC4792175

[8] Martínez-García A, Alvarez-Romero C, Román-Villarán E, et al. Fair principles to improve the impact on health research management outcomes. Heliyon. 2023;9:e15733. doi: 10.1016/j.heliyon.2023.e15733 37205991 PMC10189186

[9] Tuladhar S, Mwamelo K, Manyama C, et al. Proceedings from the CIHLMU 2022 symposium: “availability of and access to quality data in health”. BMC Proc. 2023;17:21. doi: 10.1186/s12919-023-00270-1 37587461 PMC10433535

[10] Landi A, Thompson M, Giannuzzi V, et al. The “A” of FAIR – as open as possible, as closed as necessary. Data Intel. 2020;2:47–55. doi: 10.1162/dint_a_00027

[11] World Health Organization. WHO guiding principles for pathogen genome data sharing [Internet]. Geneva: World Health Organization [cited 2025 Apr 12]. Available from: https://www.who.int/publications/i/item/9789240061743

[12] Ramsay M, Sankoh O. African partnerships through the H3Africa consortium bring a genomic dimension to longitudinal population studies on the continent. Int J Epidemiol. 2016;45:305–308. doi: 10.1093/ije/dyv187 26659658 PMC5841636

[13] Serwadda D, Ndebele P, Grabowski MK, et al. Open data sharing and the global south—who benefits? Science. 2018;359:642–643. doi: 10.1126/science.aap8395 29439233

[14] Tekola-Ayele F, Rotimi CN. Translational genomics in low- and middle-income countries: opportunities and challenges. Public Health Genomics. 2015;18:242–247. doi: 10.1159/000433518 26138992 PMC4514540

[15] Ramsay M. African genomic data sharing and the struggle for equitable benefit. Patterns (NY). 2022;3:100412. doi: 10.1016/j.patter.2021.100412 PMC8767304 35079718

[16] Pratt B, Bull S. Equitable data sharing in epidemics and pandemics. BMC Med Ethics. 2022;22:1–4. doi: 10.1186/s12910-021-00701-8 PMC8493940 34615519

[17] DS-I Africa Data Science for Health Discovery and Innovation in Africa [Internet]. DS-I Africa, Cape Town. [cited 2025 Apr 12] Available from: https://dsi-africa.org/#about

[18] Glover K, Osler T, Adetunji K, et al. Leveraging data science to understand and address multimorbidity in Sub-Saharan Africa: the MADIVA protocol. BMJ Health & Care Informatics. 2025;32:e101294. doi: 10.1136/bmjhci-2024-101294 PMC12258287 40639840

[19] Skou ST, Mair FS, Fortin M, et al. Multimorbidity. Nat Rev Dis Primers. 2022;8:48. doi: 10.1038/s41572-022-00376-4 35835758 PMC7613517

[20] Kamp M, Achilonu O, Kisiangani I, et al. Multimorbidity in African ancestry populations: a scoping review. BMJ Glob Health. 2023;8:e013509. doi: 10.1136/bmjgh-2023-013509 PMC10711865 38084495

[21] African Population and Health Research Center [Internet]. Nairobi: APHRC. [cited 2025 Apr 12]. Available from: https://aphrc.org/?gad_source=1&gclid=Cj0KCQjwhr6_BhD4ARIsAH1YdjDASrPhpn6W499cjqXwdyNIpNUbX7wj_wjRN2XpvJ2Hz976a_0bkxYaAtapEALw_wcB

[22] Protection of Personal Information Act 4 of (SA). South African Legislation; 2013.

[23] Data Protection Act, 2019 (KE). Kenyan Legislation.

[24] eLwazi Open Data Science Platform [Internet]. Cape Town: eLwazi [cited 2025 Apr 12]. Available from: https://elwazi.org/

[25] The European Genome-Phenome Archive [Internet]. UK, Spain: European Genome-phenome Archive. [cited 2025 Apr 12]. Available from: https://www.ega-archive.org/

